# Effects of Transcranial Direct Current Stimulation on Social Attention Patterns and Emotion Recognition Ability in Male Adolescents with Autism Spectrum Disorder

**DOI:** 10.3390/jcm12175570

**Published:** 2023-08-26

**Authors:** Karin Prillinger, Stefan T. Radev, Gabriel Amador de Lara, Sonja Werneck-Rohrer, Paul L. Plener, Luise Poustka, Lilian Konicar

**Affiliations:** 1 Department of Child and Adolescent Psychiatry, Medical University of Vienna, 1090 Vienna, Austria; 2 Comprehensive Center for Pediatrics (CCP), Medical University of Vienna, 1090 Vienna, Austria; 3 Cluster of Excellence STRUCTURES, Heidelberg University, 69120 Heidelberg, Germany; stefan.radev@uni-heidelberg.de; 4 Department of Child and Adolescent Psychiatry and Psychotherapy, University of Ulm, 89073 Ulm, Germany; 5 Department of Child and Adolescent Psychiatry and Psychotherapy, University Medical Center Göttingen, 37075 Göttingen, Germany

**Keywords:** autism spectrum disorder, brain stimulation, tDCS, eye tracking, social cognition, emotion recognition

## Abstract

Autism Spectrum Disorder (ASD) is characterized by impairments in social cognition including emotion recognition (ER) abilities. Common symptoms include unusual patterns of visual social attention, which are investigated as early developmental biomarkers for ASD. Transcranial Direct Current Stimulation (tDCS) has shown promising results in influencing social functioning in individuals with ASD. However, the effects of tDCS on social attention patterns and ER ability in adolescents with ASD remain unclear. This double-blind, sham-controlled, randomized clinical trial examined the effects of repeated sessions of tDCS on gaze behavior and ER ability in 22 male adolescents diagnosed with ASD. Participants received either 20 min of 2 mA active tDCS or sham stimulation for 10 days and an intra-stimulation training. Social allocation patterns were assessed using eye-tracking paradigms, including ER tasks. Our results indicated no tDCS-specific effects. Both groups showed improvements in ER and more frequent, faster, and longer fixations on the eyes than the mouth, and on social than nonsocial areas. In tasks with low social content, fixating the mouth seemed to increase ER accuracy. Understanding the effects of tDCS on social functioning in adolescents with ASD holds promise for the development of targeted interventions to improve their social cognition abilities.

## 1. Introduction

Autism spectrum disorder (ASD) is a neurodevelopmental disorder characterized by a wide range of persistent symptoms affecting reciprocal social interaction, social communication, and behavior [1]. A crucial aspect of social cognition, and therefore successful social interaction and communication, entails the identification and interpretation of emotional states from facial expressions [2,3]. This ability to recognize basic emotions from faces has repeatedly been found to be impaired in individuals with ASD [4,5]. Yet, there is controversy about whether this deficit affects the recognition of all or only some emotions [5]. A recent meta-analysis including 148 studies indicated that individuals with ASD exhibited significant and similar impairment in the recognition of all six basic emotions (fear, disgust, sadness, anger, happiness, and surprise), as well as neutral expressions [6]. Additionally, individuals with ASD were substantially worse at recognizing complex emotions (such as embarrassment or jealousy) than basic emotions. However, their general emotion recognition performance seemed not to depend on task-inherent factors such as motion (static or dynamic faces), social relevance (including the depiction of real humans or cartoons, gaze direction, and familiarity of expression), or stimulus salience (including stimulus presentation duration and emotion intensity level) [6]. Considering that familiarity [7] and emotion intensity [8] play a role in emotion recognition abilities in typically developing (TD) individuals, the absence of such impact in individuals with ASD might be attributed to the use of compensatory mechanisms, such as explicit strategies instead of automatic processing [9].

TD children acquire basic emotion recognition abilities at an early age and have an attention bias in relation to the social world (i.e., looking at other people and attending to faces) [10,11]. This bias is part of visual-social attention and can be measured using eye-tracking technology [12], and is investigated as an early developmental biomarker for ASD [13].

A meta-analysis of studies investigating eye gaze behavior in individuals with ASD found aberrant attention allocation patterns compared to TD controls, indicating a tendency to attend to social information to a lesser extent [14]. Specifically, results showed overall reduced attention to different parts of social stimuli, particularly to eyes, mouth, and the face altogether [14]. Moreover, when stimuli exhibited a high level of social content through depictions of multiple persons, the social attention of individuals with ASD was significantly reduced [11]. Yet, individuals with ASD spend more time attending to the body and non-social elements [14].

Investigating the specific circumstances under which individuals with ASD show impaired social attention, another meta-analysis found that the following factors have negligible influence: age (testing potential developmental patterns of social attention), motion (static vs. dynamic stimuli), ecological validity (e.g., drawing vs. realistic pictures), IQ, audio input, and communicative intent [11]. In accordance with diminished ability to accurately recognize complex emotions [6], eye-tracking results in adults with ASD revealed increased fixation on the mouth when presented with stimuli involving a single actor expressing a complex emotion, in contrast to the gaze pattern observed in neurotypical adults [15]. This atypical attention allocation to social stimuli has been shown to have negative effects on emotion recognition [16,17].

Although facial emotion recognition is overall worse in ASD than in other psychiatric disorders, it is important to note that there are considerable individual differences within the ASD population [6]. However, even mild impairments in facial emotion recognition can be a source of social difficulties due to the subtle, complex, and transient nature of emotional expressions that commonly occur in day-to-day social interactions [18].

Recently, transcranial direct current stimulation (tDCS) has emerged as a new approach for enhancing facial emotion recognition abilities. Encouraging results have been reported from studies conducted with diverse populations, including patients with schizophrenia and depression [19], healthy individuals [20], and adults with ASD [21]. TDCS is a noninvasive neurophysiological method that influences neuronal activity by the application of weak direct electrical currents (usually 1–2 mA) over the scalp via electrodes [22]. Affecting neuronal resting membrane potentials, anodal stimulation increases neural excitability, whereas cathodal stimulation reduces it [22]. In recent years, tDCS has been increasingly investigated as a promising and well-tolerated technique in the treatment of ASD and has shown improvement in social and behavioral symptoms as well as neuropsychological functions [23,24]. 

Different electrode montages and stimulation parameters have been proposed and investigated; based on existing evidence, the left dorsolateral prefrontal cortex (DLPFC) has been recommended as a placement site for the anodal electrode in individuals with ASD [23]. Moreover, our previous simulations have shown that this montage generates peak magnitudes at the medial prefrontal cortex (MPFC) [25]—an area responding to gaze patterns and involved in the detection of gaze direction [26,27,28]. 

Currently, there are no published studies investigating the effects of tDCS on eye gaze in individuals with ASD. However, Qiao et al. (2020) reported a neuromodulatory effect of multiple sessions of anodal high-definition tDCS on facial cognitive processing in individuals with high autistic traits. Specifically, the authors found that tDCS facilitated gaze behavior by increasing fixation duration and the number of fixations for happy and fearful faces in the mouth area [29].

Overall, in individuals with ASD, attentional and cognitive processing of emotional faces seems to follow an atypical trajectory throughout development. Although the observed deviations in eye gaze patterns are not definitively conclusive, results suggest that individuals with ASD exhibit altered visual attention toward facial emotions [30]. This atypical gaze behavior could be a contributing factor to the observed difficulty in emotion recognition [9,30] and tDCS has shown promising results in improving emotion recognition abilities and changing gaze behavior.

Thus, the goal of the current study was to systematically examine the effects of tDCS on gaze behavior and emotion recognition ability in adolescents diagnosed with ASD. Overall, we anticipated heightened attention to social areas after the tDCS intervention. Specifically, we assessed the effects of repeated tDCS sessions on gaze patterns and emotion recognition performance in four specifically designed tasks comprising both basic and complex emotions, presented in contexts with varying levels of social content. Gaze patterns were operationalized via the outcomes *Number of Fixations*, *Fixation Duration*, and *Time to First Fixation*. Accordingly, in each task, we expected tDCS-specific improvements in these outcomes (i.e., increased task performance, increased *Number of Fixations*, and lengthened *Fixation Duration* and shortened *Time to First Fixation*) as manifested by different pre–post changes between the active and the sham group.

## 2. Materials and Methods

### 2.1. Design and Participants

The study was conducted as a randomized, double-blind, and sham-controlled clinical trial. Participants were recruited from the outpatient unit at the Child and Adolescent Psychiatry department at the Medical University of Vienna as well as from local institutes and practitioners specialized in treating individuals with ASD. Inclusion criteria comprised being male, right-handed, and between 12 and 18 years, having an IQ ≥ 70, having no prior experience with neurostimulation, and fulfillment of ICD-10 criteria for ASD diagnosed by a trained professional using the Autism Diagnostic Interview-Revised (ADI-R; [31]) and/or the Autism Diagnostic Observation Schedule (ADOS 2; [32]). Participants could not be included if they met any contraindication for tDCS or magnetic resonance imaging; had epilepsy or related seizure disorders or other severe neurologic or psychiatric disorders or medical conditions; or were taking concomitant psychopharmacological medication. Due to limited evidence on drug–tDCS interaction effects [33], individuals taking medications were included in the trial only if they discontinued their psychopharmacologic medication before the baseline measures until after the last post-measurement. Concomitant long-term social or therapeutical interventions were allowed and should be continued throughout the intervention.

Interested participants and their caregivers were invited to an individual study information visit including an assessment of the medical history, and received information sheets and consent forms about the study to take home. Participants and caregivers had to return their signed informed consent forms before being included in the study. Included participants were randomly allocated to either active or sham stimulation using a script. The allocation was stratified for three conditions: a subgroup without comorbidities or based on the primary comorbidity divided into a subgroup diagnosed with depression and a subgroup diagnosed with attention-deficit/hyperactivity disorder and/or conduct disorder. To ensure a double-blind procedure, group allocation and preparation of stimulation codes were conducted by a researcher not engaged in the intervention and participant management. In the days before and after the two-week tDCS intervention, participants conducted behavioral baseline and post-measures including emotion recognition tasks and eye-tracking measures.

A total of 30 participants were screened and 23 were included in the trial. Reasons for non-participation were the impossibility of discontinuing medication in four cases, not meeting IQ criteria in two cases, and one individual mentioning the time required as a factor. The trial started in July 2019 and, due to COVID-19 restrictions, the study had to be stopped for several months in 2020. Consequently, one participant had to drop out after stimulation session four (see Figure S1 for CONSORT flow diagram). All participants were right-handed and had normal or corrected-to-normal vision. 

### 2.2. Intervention

The intervention consisted of 10 sessions of 20 min anodal tDCS or sham stimulation over two consecutive weeks. TDCS was applied at 2 mA current strength using an Eldith-DC Stimulator (NeuroConn GmbH, Germany) using 3.2 × 3.2 cm rectangular rubber electrodes and conductive Ten20 paste. The anode was placed at F3 (according to the international 10–20 system for electroencephalography) and the cathode was over the right supraorbital region. The stimulation started and ended with 30 s fade-in and fade-out phases. For the sham stimulation, 40 s of 2 mA were applied between the fade-in and fade-out phase, so participants experienced the skin sensation that is typically felt at the onset of active tDCS stimulation. During the 10 stimulation sessions, participants watched the child-friendly movie *Inside Out* [34] and performed a self-developed computer-based training targeting emotion recognition abilities, which we refer to as the emotion recognition task *(ERT).* The ERT is described in more detail as intra-stimulation training in the corresponding study protocol [25] and in the Section 2.3. The eye-tracker-based pre- and post-assessment used a different stimuli set than the intra-stimulation training version.

### 2.3. Eye Tracking Stimuli and Procedure

Participants’ gaze behavior was recorded during two paradigms. First, they performed a self-developed emotion recognition task (*ERT*). This paradigm contained three distinct labeling tasks (*Face Emotion*, *Social Scenes*, and *Morphing*) evaluating different explicit emotion recognition abilities. *Face Emotion* entailed the presentation of 18 videos featuring a single individual expressing either a basic or a complex emotion. *Social Scenes* introduced a more intricate and ecologically valid scenario, depicting eight nonverbal dynamic social interactions with emotional content involving two or more individuals. Dynamic, experimental stimuli were used in *Morphing,* which contained 42 computer-generated videos, wherein facial expressions transition from a neutral state to a basic emotional state. Participants were instructed to interrupt the morphing process as soon as they recognized the displayed emotion. In all parts, performance scores were calculated as participants had to classify the perceived emotion after each item via a mouse click on a build-in questionnaire providing six answer options (which was displayed until the participant responded). *Face Emotion* and *Social Scenes* contained complex emotions, whereas *Morphing* items showed one of the basic emotions (happy, anger, fear, disgust, sad) and neutral expressions. The emotional stimuli used in the *ERT* have been obtained from validated databases [35,36,37,38] and represent individuals of all ages and genders. To avoid order effects, the presentation order of the stimuli was pseudorandomized within the parts, using different presentation sequences in Tobii Studio [39]. 

As a second paradigm, the Movie for the Assessment of Social Cognition (*MASC)* [40,41], a valid, reliable, and sensitive measure of social cognition in adolescents, was shown to the participants. The movie had a duration of about 15 min and was interrupted 43 times to ask questions regarding the actors’ emotions, thoughts, and intentions in the last scene. The questions and answers were presented on the screen and participants gave their answers to the experimenter. Participants were instructed to verbally provide the label of the answer options presented on the screen and not ask any content-related questions to the experimenter during the measurements. From the accurate responses, the revised *MASC* score, which serves as an adolescent-adapted indicator of the level of social cognition, was calculated.

### 2.4. Eye-Tracking Apparatus

Eye-tracking data were recorded in a windowless room with constant illumination using Tobii Pro eye-tracker TX300 (Tobii Group, Danderyd, Schweden) and Tobii Studio 3.4.5 [39]. Eye movements from both eyes were captured using infrared emitters and a high-speed infrared camera positioned beneath the test screen operating at 60 Hz. Participants were seated about 60 cm from the screen displaying the stimuli and their heads were not fixated. To reduce head movement, participants were instructed to move their heads and bodies as little as possible. A separate experimenter screen was used to monitor eye movements throughout the experiments. An established 5-point calibration routine was performed before each paradigm. 

### 2.5. Classification of Fixations and Areas of Interest

**Figure 1:**
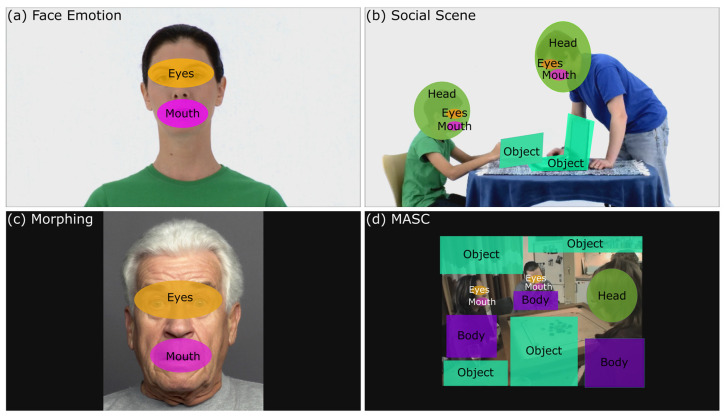
Display of areas of interest (AOIs) defined for eye-tracking data analyses. Dynamic AOIs were used for (**a**,**b**,**d**). Screenshots from the stimuli videos are illustrated: (**a**) *Face Emotion* contained AOI for eyes and mouth; (**b**) *Social Scenes* comprised AOIs for eyes, mouths, heads, and objects; (**c**) *Morphing* used static AOIs for eyes and mouths; due to restricted access to the original stimuli database, a publicly available image from the same database was utilized to illustrate the AOI definition; (**d**) MASC included AOIs for eyes, mouths, heads, bodies, and objects, which were predefined [40].

As a fixation filter, Tobii I-VT (identification-velocity threshold) was used with a velocity threshold of 30°/s to define fixations within a 20-milliseconds (ms) timeframe. Further filtering specifications consist of a minimum fixation duration of 60 ms and a gap fill-in interpolation of 75 ms. The areas of interest (AOIs) were created using Tobii Studio 3.4.5 [39]. Dynamic AOIs were defined manually for every frame of the *ERT*, based on stimuli characteristics and exemplarily shown for each task in Figure 1. For the *Social Scenes* and *MASC* tasks, AOIs were further grouped into social AOIs (e.g., eyes, mouth, head, and body) and non-social AOIs (e.g., different objects).

Depending on the stimuli specifics, different indicators were tracked as a reflection of gaze attention: *Number of Fixations* (fixation count for an AOI), *Time to First Fixation* (time from media display till participants fixate on an AOI for the first time), and/or *Fixation Duration* (sum of the duration of fixations within an AOI). Specifically, *Time to First Fixation* was calculated for the *Morphing* and *Face Emotion* tasks, as only in these tasks did all relevant AOI occur from the beginning of the media display. *Fixation Duration* was assessed for the *Face Emotion*, *Social Scenes,* and *MASC* tasks. It was not calculated for the *Morphing* task, as participants could individually interrupt this task and, therefore, a comparison of *Fixation Duration* would not be valid. *Fixation Duration* was calculated as the total sum of individual fixation durations per AOI (e.g., eyes, object) in each trial. Also, the accuracy of emotion recognition was measured in all tasks.

### 2.6. Statistical Analysis

We conducted all statistical analyses using R, Version 4.1.3 [42]. For each of the three tasks of the *ERT* paradigm, that is, *Morphing*, *Social Scenes*, and *Face Emotion*, as well as the *MASC* paradigm, we fit linear mixed-effects models for analyzing the participants’ *trial-by-trial* performance and eye gaze behavior. The advantages of using single-trial mixed-effects models are that they can naturally deal with nested observations (e.g., repeated measurements) and different numbers of trials per participant (e.g., due to absence). Moreover, they do not ignore data variability through aggregation (e.g., averaging over trials) and automatically regularize the influence of outliers (i.e., shrinkage). We applied the lme4 package [43] to fit the models, which we describe below using Wilkinson notation.

**Models of task performance.** We analyzed the participants’ emotion recognition performance in each task by considering their trial-by-trial accuracy. The single-trial model of accuracy is thus given by:

$$Accuracy\;\sim \;Time*Group+\left(Time\;\right|\;ID)+(1\;\left|\;Item\right)$$

where we apply the *logit* link function (i.e., as in standard logistic regression) to account for the fact that the dependent variable can take only two values in a given trial (“correct” vs. “incorrect”). The random effects factor *Item* was replaced by *Emotion* for the *Morphing* task.

**Models of gaze behavior.** For the *Morphing* and *Face Emotion* tasks, we first analyzed the participants’ *Fixation Rates* through a binary indicator of occurrence or non-occurrence of a fixation in a given trial. The *Fixation Rate* indicator provided valuable information since non-fixations are themselves an indicator of behavior and may co-vary with the experimental design factors. The logistic model for the fixation indicator variable is thus:

$$Fixation\;\sim \;Time*Group*AOI+\left(Time\;\right|\;ID)+(1\;\left|\;Item\right)$$

Second, we analyzed the trial-by-trial *Time to First Fixation* (TTFF) as one of our primary eye-tracking dependent variables operationalizing gaze behavior. Thus, we specified the following models of TTFF for the *Morphing* and *Face Recognition* tasks:

$$TTFF\;\sim \;Time*Group*AOI+\left(Time\;\right|\;ID)+(1\;\left|\;Item\right)$$

where once again the random effects factor *Item* in both of the above models was replaced by *Emotion* for the *Morphing* task.

For the *Face Emotion*, *Social Scenes*, and *MASC* tasks, we analyzed the *Number of Fixations* in a given AOI within each trial. We expect this variable to be highly correlated with *Fixation Duration*, yet to also provide incremental information, since fixation duration may not be uniquely determined by fixation frequency (and vice versa). Thus, we formulated the following models of trial-by-trial *Number of Fixations* (*#Fixations*) and *Fixation Duration* (*FD*):

$$\#Fixations\;\sim \;Time*Group*AOI+\left(Time\;\right|\;ID)+(1\;\left|\;Item\right)$$

$$FD\;\sim \;Time*Group*AOI+\#Fixations+\left(Time\;\right|\;ID)+(1\;\left|\;Item\right)$$

In line with previous literature including the *Number of Fixations* in the analysis [17,44], we control for the *Number of Fixations* when modeling Fixation Duration.

**Auxiliary analyses.** In order to relate task performance and gaze behavior, we either included the binary fixation indicators (*Morphing* and *Face Emotion*) or the *Number of Fixations* per AOI (*Social Scenes and MASC*) as additional covariates in each logistic model of performance. Since we treated these analyses as exploratory, we augmented each task performance model in a data-driven manner based on the results of the eye gaze models.

**Model choice.** To assess the effects of the tDCS intervention on emotion recognition performance and gaze behavior, we focused on the *time* × *group* interaction in each of the models. We consider interaction effects statistically significant if they do not surpass the standard Type I error threshold of *α* = 0.05. Additionally, we also performed a likelihood-ratio test between the *full model* and a *reduced (nested) model* including no *group* factor; we only selected the full model if a significant likelihood ratio test was confirmed by the AIC and BIC criteria. Due to the exploratory nature of these analyses of eye-tracking variables, which did not comprise the primary outcomes described in the study protocol [25], and the consideration of multiple model fit criteria, we did not correct for multiple comparisons.

## 3. Results

For the analysis, the 22 participants (mean age 14.1 ± 1.9 years) who received all 10 tDCS sessions were included. The duration of the paradigms and performance scores of both groups and time points are presented in Table 1.

In the following, we describe the results of our comprehensive analyses for each of the *ERT* tasks. The inclusion of random effects was warranted for all models, as measured by considerable gains in adjusted explained variance ratios (*R^2^*) and standard model comparison metrics (e.g., AIC, BIC). Thus, the results focus exclusively on the mixed-effects models. For all analyses, we report either the results of the reduced or the full model, depending on which model was preferred by the model selection criteria. Complete model results, as well as additional checks, are provided in the supplementary material. 

### 3.1. Morphing Task

**Figure 2:**
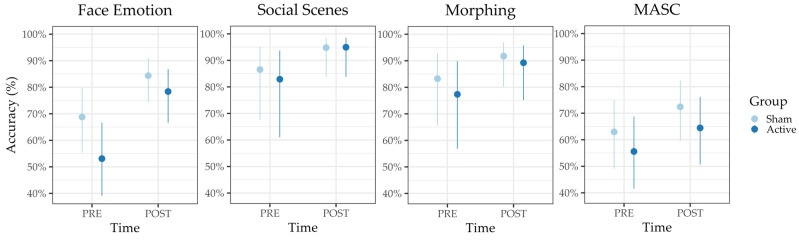
Model predictions for the average accuracy per group over time of emotion recognition performance showed significant improvement over time for both groups in all four tasks. Bars represent confident intervals of the predicted mean.

**Task performance.** The winning reduced model (i.e., excluding the group factor) revealed a significant main effect of time (OR = 2.24, 95%-CI [1.73–3.15], *p* < 0.001). Accordingly, participants in both groups significantly improved their accuracy over the course of the ER training (see Figure 2). Finally, the reduced model estimated a small correlation, r = 0.31, between random intercepts and random slopes.

**Figure 3:**
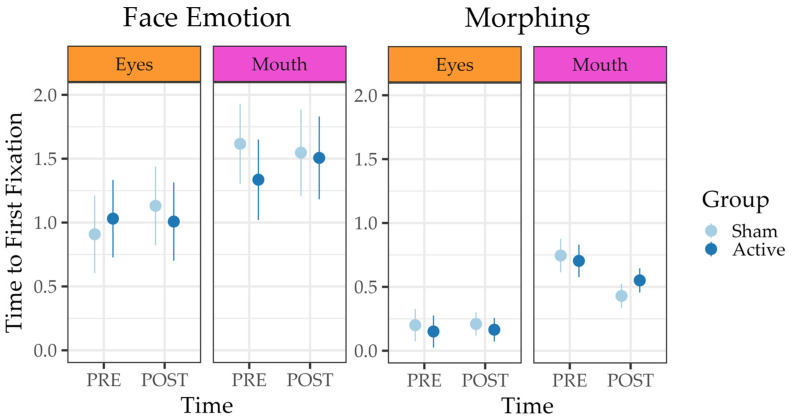
Model predictions for the average *Time to First Fixation* in the *Morphing* and *Face Emotion* tasks implied that participants tended to fixate the eyes prior to the mouth at both time points. In the *Morphing* task, participants in both groups fixated on the mouth significantly faster after the intervention. Bars represent 89% confidence intervals of the predicted mean.

**Figure 4:**
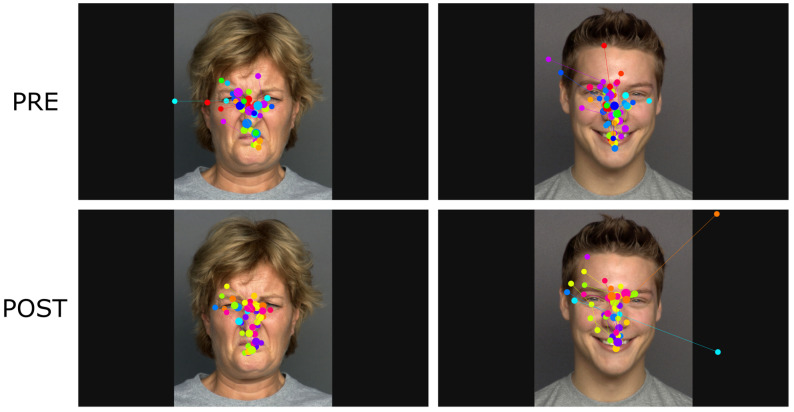
Gaze plots illustrating gaze patterns on two example stimuli expressing different emotions from the *Morphing* task at pre and post collectively for all participants. The positions of fixations are represented as dots, with the size of the dots indicating the duration of the fixations. As participants interrupt the Morphing phase upon emotion recognition, gaze plots visualize gaze behavior up until the mean *Time to First Fixation* on the mouth AOI (0.836 s) for both groups. Due to restricted access to the stimuli database, gaze behavior was exported and superimposed on a publicly available image from the same database using images with matching characteristics regarding age, gender, and emotion.

**Gaze behavior.** The winning reduced model of *Fixation Rate* revealed a significant effect of AOI (OR = 0.26, 95%-CI [0.20–0.33], *p* < 0.001) and a significant interaction between time and AOI (OR = 1.45, 95%-CI [1.45–2.02], *p* = 0.028). Finally, the model estimated a large negative correlation of r = −0.84 between random intercepts and slopes. As for the TTFF, the preferred full model revealed a significant main effect of AOI (β = 0.54, 95%-CI [0.48–0.61], *p* < 0.001), a significant two-way interaction between time and AOI (β = −0.33, 95%-CI [−0.42–−0.23], *p* < 0.001), and a significant three-way interaction between time, AOI, and group (β = 0.16, 95%-CI [0.03–0.28], *p* < 0.013). The results are illustrated in Figure 3 and Figure 4. Finally, the model estimated a large negative correlation of r = −0.77 between random intercepts and slopes. The three-way interaction is illustrated in Figure 3 and implies that on average, participants tended to fixate on the “eyes” AOI prior to the “mouth” AOI. Following the intervention, participants tended to fixate the “mouth” AOI significantly faster (albeit still slower than the “eyes” AOI), especially in the sham group. 

**Auxiliary analysis.** Based on the results regarding differences between the *Fixation Rates* of the two AOIs, we augmented the reduced model of task performance (i.e., featuring no group factor) with two additional variables indicating whether in a given trial the “mouth” AOI and/or the “eyes” AOI were fixated on or not. This model revealed a significant interaction between the *Fixation Rate* for the “mouth” AOI and time, (OR = 3.73, 95%-CI [1.13–12.13], *p* = 0.029). The interaction between the *Fixation Rate* for the “eyes” AOI and time showed a similar trend; however, it barely missed the significance threshold, (OR = 2.59, 95%-CI [0.97–6.90], *p* = 0.058).

### 3.2. Face Emotion Task

**Task performance.** The selected reduced model of accuracy (i.e., without the group factor) revealed a significant main effect of time, (OR = 2.82, 95%-CI [2.00–3.98], *p* < 0.001), see Figure 2. Since the random slopes model failed to converge, these results are based on a random-intercept-only model and no correlation between random intercepts and slopes could be estimated.

**Gaze behavior.** First, the selected reduced model of Number of Fixations yielded a significant main effect of AOI, (β = −0.56, 95%-CI [−0.64–−0.48], *p* < 0.001). The random slope estimate suggested negligible variability.

**Figure 5:**
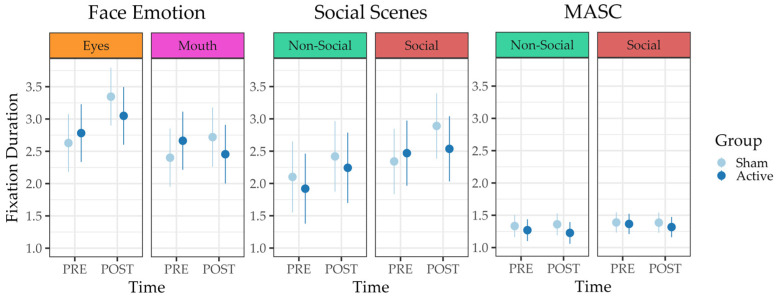
Model predictions of average *Fixation Duration* in the *Face Emotion*, *Social Scenes*, and *MASC* tasks. Bars represent 89% confidence intervals of the predicted mean. In the *Face Emotion* task, we found an increased *Fixation Duration* in the eyes area for both groups. In the *Social Scenes* task, we found an overall increase in *Fixation Duration* in the social areas (i.e., eyes, mouth, head) for both groups. In the *MASC* paradigm, an overall higher *Fixation Duration* in the social areas than in the non-social ones was observed.

**Figure 6:**
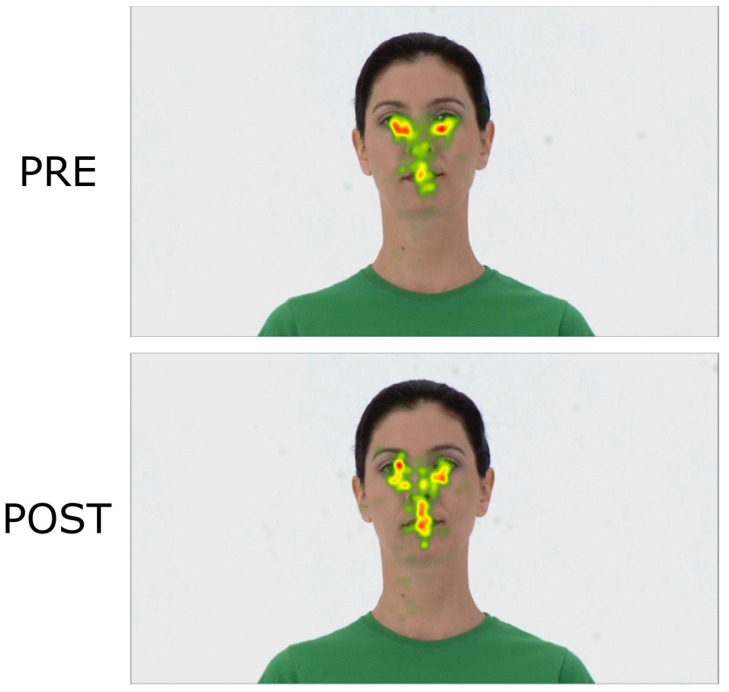
Heat maps illustrating fixation duration on a representative stimulus from the *Face Emotion* task for all participants. The accumulated *Fixation Duration* at pre and post is depicted, providing a visualization of the attention dedicated to different features in the stimuli. Each participant’s fixation contributes color-coded information based on their respective duration (in seconds). The color scale ranges from red (indicating the longest fixations) to green (indicating the shortest fixations), with intermediate colors representing varying durations.

Second, the winning full model of Fixation Duration yielded a significant main effect of time, (β = 0.72, 95%-CI [0.49–0.94], *p* < 0.001), a significant main effect of the (scaled and mean-centered) Number of Fixations, (β = 1.66, 95%-CI [1.60–1.73], *p* < 0.001), a significant time*AOI interaction (β = −0.40, 95%-CI [−0.74–0.06], *p* = 0.021), and a significant time*group interaction, (β = −0.45, 95%-CI [−0.77–−0.13], *p* = 0.006). This rather involved result is illustrated in Figure 5. The random slope estimate was negligible. The effect of Fixation Duration on time*AOI is shown in Figure 6.

Third, the preferred reduced model of TTFF revealed only a significant main effect of AOI, (β = 0.51, 95%-CI [0.27–0.74], *p* < 0.001), suggesting a considerably longer overall TTFF for the “mouth” AOI (see Figure 3). Once again, the random slope estimate was negligible. This result is paralleled by the reduced logistic model of *Fixation Rate*, which also yielded a sole significant main effect of AOI, (OR = 0.19, 95%-CI [0.10–0.36], *p* < 0.001).

**Auxiliary analysis.** To relate task performance to gaze behavior, we augment the reduced model of accuracy by two further main effects signifying whether in a given trial the “mouth” AOI and/or the “eyes” AOI were fixated on. This model reproduced the significant effect of time, (OR = 2.72, 95%-CI [1.95–3.80], *p* < 0.001), and additionally estimated a significant effect of *Fixation Rate* in the “mouth” AOI (OR = 1.87, 95%-CI [1.22–2.85], *p* = 0.004).

### 3.3. Social Scenes Task

**Task performance.** A significant effect of time, (OR = 3.31, 95%-CI [1.68–6.53], *p* < 0.001) emerged from the preferred reduced model of accuracy (see Figure 2), which used only a random intercept due to convergence issues.

**Gaze behavior.** The analysis of (log) Number of Fixations indicated no preference for the full model and no significant predictors in either of the models. Thus, we performed no auxiliary analyses. The preferred full model of Fixation Duration revealed a significant main effect of the (scaled and mean-centered) Number of Fixations, (β = 0.56, 95%-CI [0.54–0.57], *p* < 0.001), a significant two-way interaction between time and AOI, (β = 0.10, 95%-CI [0.01–0.20], *p* = 0.035), along with a significant three-way interaction between time, AOI, and group, (β = −0.16, 95%-CI [−0.30–−0.03], *p* = 0.019). This result is illustrated in Figure 5. The model features only a random intercept since it failed to converge otherwise.

### 3.4. MASC

**Task performance.** *MASC-R* contains six control questions to control for understanding and attention during the paradigm, which are not included in the overall score. None of the participants answered all of the control questions wrong, and therefore, all *MASC-R* scores were included in the analysis. The preferred reduced model estimated that the sole predictor of accuracy was *time*, (OR = 1.49, 95%-CI [1.21–1.85], *p* < 0.001), see Figure 2.

**Gaze behavior.** The preferred reduced model of Fixation Duration estimated a sole significant main effect of AOI (β = 0.28, 95%-CI [0.25–0.32], *p* < 0.001), implying that “social” AOIs were fixated on more frequently than “non-social”. This result was paralleled by the reduced model of Fixation Duration, which also estimated a significant, yet smaller, main effect of AOI (β = 0.08, 95%-CI [0.02–0.14], *p* = 0.013), even when controlling for the (scaled and mean-centered) Number of Fixations (β = 1.68, 95%-CI [1.67–1.70], *p* = 0.001) (see Figure 5).

**Auxiliary analysis.** Finally, to relate task performance to gaze behavior in the MASC paradigm, we augmented the reduced model of accuracy by two further main effects denoting the average Number of Fixations in the “social” and “non-social” AOIs, respectively. This model replicated the significant effect of time (OR = 1.51, 95%-CI [1.22–1.88], *p* < 0.001), and additionally revealed a significant effect of the average *Number of Fixations* in the “non-social” AOI (OR = 1.08, 95%-CI [1.02–1.14], *p* = 0.004).

## 4. Discussion

This is the first randomized, double-blind, and sham-controlled clinical trial to investigate the effects of multiple sessions of tDCS on emotion recognition ability and gaze behavior in adolescents with ASD. Below we summarize and discuss the main findings of the study.

### 4.1. Emotion Recognition Performance

We found no intervention-specific improvements in emotion recognition in the *ERT (Morphing*, *Social Scenes*, *Face Emotion)* and *MASC* paradigms. However, participants in both groups improved their accuracy in all tasks following the intervention.

A recent meta-analysis found that the recognition of basic and complex emotions is impaired in ASD [6]. Our results showed an improvement in accuracy in the recognition of basic and complex emotions, involving experimental and naturalistic stimuli as well as stimuli with low (one person) and high (up to four persons interacting) social content. Therefore, the intra-stimulation training might have exerted an effect on the current *ERT* results. Moreover, the training effect may also have generalized to more complex social cognition tasks, like the *MASC*. Our sample had a mean *MASC-R* score at baseline (group-independent 22.91) similar to the validation study, in which the sample of adolescents with ASD scored 23.6 points. However, the *MASC-R* score was higher after the 10-day intervention (group-independent 26 points) but was still below the TD controls, which reached a score of 29.2 points [40]. The duration of *MASC* was also slightly shorter (32.8 min) than the baseline duration recorded in this study (36.2 min).

Interestingly, in the Morphing task, which consists of low social content and basic emotion stimuli, participants who achieved higher accuracy pre-training tended to show a slightly greater improvement. This might indicate that in comparably easier tasks, some baseline ability is necessary to successfully train the emotion recognition ability.

### 4.2. Fixation Rates, Number of Fixations, and Influence on Emotion Recognition Performance

Regarding *Fixation Rates* and *Number of Fixations*, independently of the intervention, participants in both groups tended to look at the mouth less frequently than at the eyes at both time points in the *Morphing* and *Face Emotion* tasks. This implies that in several trials, participants only looked at the eyes. Moreover, participants who were least likely to look at the eyes or mouth at baseline increased their *Fixation Rates* the most. Also, the fixations on the mouth and eyes changed differently throughout the training, with increased looking at the mouth and slightly fewer fixations on the eyes. Finally, in the *MASC*, social areas (i.e., eyes, mouth, head, and body) were fixated more often than the non-social areas (i.e., different objects). 

In line with our results, previous studies using static stimuli depicting basic emotions showed that individuals with ASD had more fixations on the eyes compared to the mouth, and therefore seem to rely on information from the eye areas. However, these studies found that participants tended to attend more to the mouth and less to the eyes when the presented stimuli displayed a complex emotion [45,46].

Interestingly, in the *Morphing* task, emotion recognition performance increased more when participants started fixating on both mouth and eyes, but fixating on the mouth seems to be more important to increase accuracy. Similarly, in the *Face Emotion* task, fixating on the mouth seems to increase the probability of correctly identifying the displayed emotion. However, in the *Face Emotion* task, participants did not learn to fixate on the mouth more often following the training, which they learned in the Morphing task. Surprisingly, in the *MASC* paradigm, the *Number of Fixations* on non-social areas was associated with a slightly increased recognition accuracy. 

Kliemann et al. (2010) found that compared to healthy controls, individuals with ASD exhibited a reduced preference for the eye area by gazing away from the eyes to the mouth more often. The authors assumed that if individuals with ASD redirect their gaze toward the mouth due to its greater informational value, prolonged *Fixation Duration* on the mouth would enhance emotion recognition performance. Surprisingly, their findings showed that a lower *Number of Fixations* on the mouth corresponded to a higher accuracy in classifying emotions [16]. Contrary to their findings, but in line with their theory, we found that an increase in fixations at the mouth is predictive of improvements in emotion recognition. A meta-analysis also reported that with increasing age, a mouth-compensation strategy develops in individuals with ASD, indicating they rely more on the mouth to retrieve social information [47]. 

### 4.3. Time to First Fixation

Regarding the TTFF in the *Morphing* and the *Face Emotion* tasks, we found that participants in both groups tended to fixate on the eyes prior to the mouth at both time points. However, only in the *Morphing* task, participants in both groups tended to fixate on the mouth significantly faster (albeit still slower than the eyes area) after the intervention. Also, participants with larger TTFFs at baseline reduced their TTFF the most. This result is consistent with previous studies conducted on individuals with ASD [45], as well as research on gaze patterns in TD controls, which has shown a preference for looking at the eyes regardless of scene complexity, including action and social content [48]. 

Interestingly, the TTFF is shorter in the speed–accuracy task *Morphing*, in which participants had to stop the Morphing process as soon as they recognized the emotion, than in the non-time-sensitive task *Face Emotion.*

### 4.4. Fixation Duration

We investigated *Fixation Duration* in the *Face Emotion* and *Social Scenes* tasks, as well as in the *MASC* paradigm. In all three tasks, a higher *Number of Fixations* was associated with a longer *Fixation Duration*. In the *MASC* paradigm, we found a higher *Fixation Duration* in the social areas than in the non-social ones for both groups, even when controlling for the *Number of Fixations*.

Müller et al. (2016) found that a longer *Fixation Duration* on eyes was associated with an increased *MASC* score and that no further AOI had a significant influence on task performance. However, our results suggest that the *Number of Fixations* in non-social areas was associated with a slightly increased recognition accuracy. Even though we grouped the AOIs for eyes, mouth, head, and body together as social areas in order to utilize all information from the task, this result was contrary to previous studies and results from our other tasks. However, this task comprised the highest social content (including several persons interacting), and the social attention of individuals with ASD is known to be reduced in social scenes with high social content (Chita-Tegmark, 2016b). On the contrary, in TD individuals, the attention to the eyes increased when stimuli had a high social content (i.e., more people), and attention to the eyes (*Number of Fixations* and *Fixation Duration*) increased even more when there was activity (i.e., the interaction of several people) in social scenes [48]. 

In the *Face Emotion* task, we found an increased *Fixation Duration* in the eye area for both groups. In the sham group, *Fixation Duration* increased in both AOIs. Correspondingly, in the *Social Scenes* task, we found an overall increase in *Fixation Duration* in the social area (i.e., eyes, mouth, and head) for both groups, which was especially pronounced in the sham group. While we found an intervention-specific effect on *Fixation Duration* for the sham group, a study by Qiao et al. (2020) reported that active tDCS facilitated gaze behavior by increasing *Fixation Duration* and *Number of Fixations* in the mouth area in individuals with high autistic traits. However, this effect was only found for happy and fearful faces, but not for neutral expressions. Also, they reported no significant changes regarding *Fixation Duration* and *Number of Fixations* on the eyes AOI in either group. In the study by Qiao et al. (2020), HD-tDCS was applied for 5 days to university students with high autistic traits. Therefore, neither the sample, the tDCS parameters (ring montage HD-tDCS over temporoparietal junction (TPJ) vs. bipolar anodal tDCS over DLPFC), the number of stimulation sessions, nor the intra-stimulation activity (no task vs. emotion recognition training) is comparable with our study. Also, their eye-tracking task was designed as a free-viewing task, which does not demand labeling and explicit emotion recognition and might result in a different activation of brain areas [49]. Another study, investigating the effects of magnetic stimulation on individuals with autism-like traits, reported increased accuracy for facial emotion recognition after active and sham stimulation. However, *Fixation Duration* on the eyes did not change significantly after the stimulation, leading to the assumption that the improvement of accuracy was not affected by longer attention to the eyes. Furthermore, the authors state that normalizing gaze behavior might be more difficult than disrupting it [50]. 

### 4.5. General Discussion, Strengths, and Limitations

Whereas current literature suggests that stimulation over the DLPFC is effective for changing ASD-related symptoms [23], regarding the improvement of emotion recognition abilities, the TPJ appears to be a promising stimulation target [20]. TDCS over the TPJ has been shown to enhance emotion recognition abilities in healthy adults [51]. However, two studies investigating the effects of tDCS over the TPJ in adults with ASD did not find significant differences in facial emotion recognition [21] and social skills [52]. Yet, significant effects on emotion recognition have been observed with tDCS over the DLPFC [19]. However, more studies are needed to investigate the effects of tDCS on gaze patterns in patients with abnormal social attention, such as individuals with ASD.

Another important aspect to consider in neurophysiological treatment studies, especially when investigating children and adolescents, is brain development [53,54]. Research on the MPFC, which is an important part of the social brain and stimulated in this study, showed that the MPFC contributes differently to mentalization tasks (e.g., emotion recognition, Theory of Mind) at different ages [25,55]. Specifically, fMRI studies showed a decrease in activation in the MPFC during the performance of mentalization tasks between adolescence and adulthood [55,56]. Moreover, studies revealed that the MPFC exhibits an alteration in functional connectivity with other brain regions with age [57]. These findings also indicate that mentalizing, which is impaired in ASD [58,59], continues to develop in adolescence and that adolescence emerges as a crucial period for the maturation of brain regions implicated in social cognition in general [55,60]. This state of structural and functional brain development during adolescence offers an opportunity to intervene with a neurophysiological intervention, such as tDCS.

Within the existing literature on ASD, there is a lack of consensus regarding social attention deficits measured using eye-tracking paradigms. This lack of consensus may be attributed to several factors, including the variability, adequacy, and sensitivity of the employed eye-tracking paradigms, as well as the unclear origin of emotion recognition deficits observed in individuals with ASD [17]. These factors, together with great variability in the assessments to examine changes in emotion recognition (for review see [18]), contribute to the heterogeneity of results observed across studies investigating social attention and emotion recognition deficits in individuals with ASD.

Compared to other studies, our paradigms included stimuli comprising complex emotions, high social content, and non-social elements, which are characteristics making emotion recognition especially difficult for individuals with ASD. Furthermore, the intra-stimulation training might have led to the improvement in both intervention groups and hidden a possible tDCS effect.

In the analysis, we considered that missing data points, such as no fixations in the eyes AOI, could be part of atypical social attention in ASD, and used trials-based instead of aggregated data. As potential limitations of this study, it is important to note that our sample consists exclusively of male adolescents without intellectual disabilities. Furthermore, our sample size was limited, and, thus, this study should facilitate mainly the generation of hypotheses for further trials. Future studies should aim to include larger and more diverse samples to enhance the validity of the results. Due to our task design, we cannot make conclusions about whether more and shorter fixations or fewer and longer fixations improve emotion recognition accuracy. It might be important to investigate this in future studies, as a longer fixation duration following an intervention may be associated with more explicit processing and therefore could be a starting point for eye-tracking-based interventions for individuals with ASD.

## 5. Conclusions

This randomized, double-blind, and sham-controlled clinical trial investigated the effects of multiple sessions of tDCS on gaze behavior and emotion recognition ability in male adolescents with ASD. Emotion recognition ability increased in both groups and generalized to a more complex paradigm comprising characteristics that are difficult for individuals with ASD. In tasks using stimuli with low social content and basic as well as complex emotions, more fixations on the mouth seemed to increase emotion recognition performance. In these tasks, as well as in tasks with high social content, participants fixated more frequently, faster, and longer at the eyes than the mouth, and more at social areas than nonsocial areas, which is in line with results for TD controls. However, while both groups improved and seemed to exhibit different eye gaze patterns after the intervention, we did not find specific improvements in the active tDCS group. Further research is needed to elucidate the effects of tDCS on atypical gaze patterns in individuals with ASD.

## Figures and Tables

**Table 1:** Duration and performance in the paradigms and age for the active and sham tDCS groups.

	Active				Sham			
	Pre		Post		Pre		Post	
	Mean	SD	Mean	SD	Mean	SD	Mean	SD
Age	14.00	1.90	-	-	14.27	1.90	-	-
ERT duration	19.17	5.05	14.43	2.63	19.06	4.38	13.85	2.05
MASC duration	35.48	4.44	31.3	4.53	36.96	5.31	34.12	9.54
Morphing score	30.18	6.40	34.91	5.34	32.82	2.52	36.64	3.38
Social Scenes score	6.00	2.37	7.09	1.22	6.45	0.93	7.27	0.65
Face Emotion Score	9.55	3.27	13.45	3.14	12.00	2.14	14.64	1.12
MASC-R score	21.91	7.73	24.73	8.05	23.91	6.41	27.27	5.66

Movie for the Assessment of Social Cognition (MASC), emotion recognition task (ERT).

## Data Availability

The data presented in this study are available on request from the corresponding author.

## Supplementary Materials

The following supporting information can be downloaded at: https://www.mdpi.com/article/10.3390/jcm12175570/s1, Figure S1: Consort Flow Diagram.
