# Supplementary material for: Effects of Transcranial Direct Current Stimulation on Social Attention Patterns and Emotion Recognition Ability in Male Adolescents with Autism Spectrum Disorder
Source: J Clin Med. 2023 Aug 26;12(17):5570. doi: 10.3390/jcm12175570 (PMC10489141; doi:10.3390/jcm12175570)
Supplement: Supplementary file 1 [file jcm-12-05570-s001.zip › jcm-2489119-supplementary.pdf]

Supplement to the manuscript "Effects of Transcranial Direct Current Stimulation on Social Attention Patterns and Emotion Recognition Ability in Male Adolescents with Autism Spectrum Disorder"

CONSORT 2010 Flow Diagram

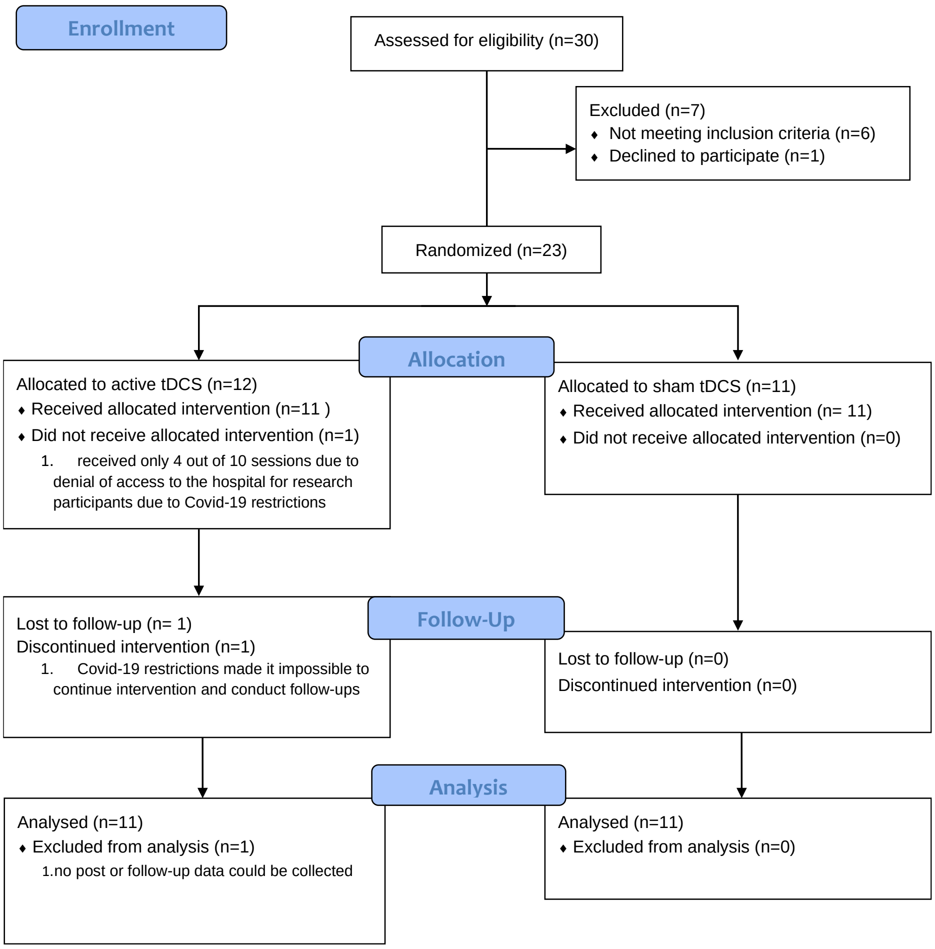

Figure S1: Consort Flow Diagram

Extended Description of Statistical Analysis

We conducted all statistical analyses using R, Version 4.1.3 (R Core Team, 2022). For each of the three tasks of the *ERT* paradigm, that is, *Morphing, Social Scenes,* and *Face Emotion,* as well as the *MASC* paradigm, we fit linear mixed-effects models for analyzing the participants' *trial-by-trial* performance and eye gaze behavior. The advantages of using single-trial mixed-effects models are that they can naturally deal with nested observations (e.g., repeated measurements) and different numbers of trials per participant (e.g., due to missingness). Moreover, they do not ignore data variability through aggregation (e.g., averaging over trials) and automatically regularize the influence of outliers (i.e., shrinkage). We apply the lme4 package (Bates, Mächler, Bolker, & Walker, 2015) to fit the models, which we describe below using Wilkinson notation.

All models feature the fixed effect factors *Time*, which indicates the measurement occasion ("pre-treatment" vs. "post-treatment") and *Group*, indicating the group assignment ("active" vs. "sham"). The models of gaze behavior include an additional fixed effect factor *AOI* which differentiates between the levels "mouth" vs. "eyes" for the *Morphing* and *Face Emotion* tasks and "social" vs. "non-social" for the *Social Scenes* and *MASC* tasks. Furthermore, all models include the participant identifier *ID* and the stimulus identifier *Item* as random effect factors to account for the nested structure of the single-trial data sets. Whenever possible (i.e., non-singular fit), we estimate random slopes along the random intercepts per participant in order to analyze the correlation between random slopes and random intercepts as an additional source of information.

Models of task performance. We analyze the participants' emotion recognition performance in each task by considering their trial-by-trial accuracy. The single-trial model of accuracy is thus given by

$$Accuracy \sim Time * Group + (Time \mid ID) + (1 \mid Item)$$

where we apply the *logit* link function (i.e., as in standard logistic regression) to account for the fact that the dependent variable can take only two values in a given trial ("correct" vs. "incorrect"). As mentioned above, the random effects factor *Item* is replaced by *Emotion* for the *Morphing* task.

Models of gaze behavior. For the *Morphing* and *Face Emotion* tasks, we first analyze a basic binary variable denoting whether a participant fixated a given AOI or not within a trial. This variable, which is essentially a proxy of a participant's "fixation rate", provides valuable information, since non-fixations are themselves an indicator of behavior and may co-vary with the experimental design factors. Moreover, this variable allows us to analyze trials for which the *Time to First Fixation* (TTFF) dependent variable is missing, since in that case the *Fixation* variable takes on a value of zero, which will be considered in the fixation rate. The logistic model for the fixation indicator variable is thus:

$$Fixation \sim Time * Group * AOI + (Time \mid ID) + (1 \mid Item)$$

where we again use the *logit* link function to account for the fact that *Fixation* is a binary indicator.

Second, we analyze the trial-by-trial TTFF as one of our primary eye tracking dependent variables operationalizing gaze behavior. Thus, we specify the following models of TTFF for the *Morphing* and *Face Recognition* tasks:

$$TTFF \sim Time * Group * AOI + (Time \mid ID) + (1 \mid Item)$$

where once again the random effects factor *Item* in both of the above models is replaced by *Emotion* for the *Morphing* task.

Additionally, for the *Face Emotion*, *Social Scenes,* and *MASC* tasks, we analyze the *Number of Fixations* in a given AOI within each trial. We expect this variable to be highly correlated with *Fixation Duration* (i.e., our second primary eye tracking dependent variable), yet to provide incremental information, since fixation duration may not be uniquely determined by fixation frequency (and *vice versa*). Thus, we formulate the following models of trial-by-trial *Number of Fixations* (*#Fixations*) and *Fixation Duration* (*FD*):

$$\#Fixations \sim Time * Group * AOI + (Time \mid ID) + (1 \mid Item)$$

$$FD \sim Time * Group * AOI + \#Fixations + (Time \mid ID) + (1 \mid Item)$$

In line with previous literature relying on aggregate analysis, we control for the *Number of Fixations* when modeling fixation duration.

Auxiliary analyses. In order to relate task performance and gaze behavior, we either include the binary fixation indicator (*Morphing* and *Face Emotion*) or the *Number of Fixations* per AOI (*Social Scenes and*

MASC) as additional covariates in each logistic model of performance. Since we treat these analyses as exploratory, we augment each task performance model in a data-driven manner: If the eye tracking variable exhibits no interaction with *Time* in the main analysis (i.e., does not exhibit a pre-post change), we only include its main effect, otherwise we include both its main effects and interactions with other fixed effect factors. The choice of eye tracking variables to include in the performance analysis is guided by the fact that these variables incorporate missingness as part of the behavior and thus lead to no loss of data points. In contrast, *TTFF* or *Fixation Duration* are only present in the case of at least one fixation within an *AOI*.

For each of the models, we test whether the inclusion of random effect factors is warranted by performing a standard likelihood ratio test between the full mixed effects model and the corresponding fixed-effects-only model. Further, to assess the effects of the tDCS intervention on emotion recognition performance and gaze behavior, we focus on the *Time***Group* interaction in each of the models. We consider interaction effects statistically significant if they do not surpass the standard Type I error threshold of $\alpha = 0.05$. Additionally, we also perform a likelihood-ratio test between the full model and a reduced (nested) model including no *Group* factor; we only select the full model if a significant likelihood ratio test is confirmed by the AIC and BIC criteria. In addition, we analyze the correlation between random slopes and random intercept to determine relationships between baseline behavior and change. Numeric variables (e.g., *Number of Fixations*) were log-transformed to reduce the effect of large positive skewness.

Full model results:

Morphing Task

Performance. The likelihood ratio test between the full model of accuracy and the model excluding the Group factor indicated no preference for the full model, $\chi2$ (2) = 1.49, p = .48. In addition, the *Time***Group* interaction in the full model did not yield a significant Odds Ratio (OR = 1.09, 95%-CI [0.61 – 1.94], p = 0.775).

Gaze behavior. The likelihood ratio test between the full model of fixation rate and the model excluding the Group factor indicated no preference for the full model, $\chi2$ (4) = 6.64, p = .16. In addition, none of the interaction terms in the full model including the *Group* factor reached statistical significance. For the model of *Time to First Fixation*, the likelihood ratio test indicated a strong preference for including the Group factor, $\chi2$ (4) = 15.73, p = .003.

Face Emotion Task

Performance. The likelihood ratio test between the full model of accuracy and the model excluding the *Group* factor indicated no preference for the full model, $\chi2$ (2) = 3.90, p = .14. In addition, the *Time***Group* interaction in the full model did not yield a significant Odds Ratio, (OR = 1.31, 95%-CI [0.66 – 2.58], p = 0.436).

Gaze behavior. Focusing on the analysis of (log) *Number of Fixations* in the different *AOI,* the likelihood ratio test between the full model and the one without the *Group* factor yielded a non-significant result, $\chi2$ (4) = 4.56, p = 0.335. The analysis of *Fixation Duration* yielded a significant likelihood ratio test, $\chi2$ (4) = 16.83, p = 0.002, between the full and the reduced model, suggesting that the inclusion of the *Group* factor is warranted. The analysis of *Time to First Fixation (TTFF)* yielded a non-significant likelihood ratio test between the full and the reduced model, $\chi2$ (4) = 3.25, p = 0.517, suggesting the redundancy of the *Group* factor for predicting *TTFF*.

Social Scenes Task

Performance. The likelihood ratio test between the full and the reduced model of accuracy yielded a non-significant result, $\chi2$ (2) = 0.28, $p = 0.869$.

Gaze behavior. The analysis of Fixation Duration found strong support for the model including the Group factor, $\chi2$ (4) = 17.84, $p < 0.001$.

MASC

Performance. The likelihood ratio test between the full and the reduced model yielded a non-significant result, $\chi2$ (2) = 0.93, $p = 0.63$. In addition, the *Time*Group* interaction in the full model did not yield a significant Odds Ratio, (OR = 0.94, 95%-CI [0.61 – 1.44], $p = 0.774$).

Eye gaze behavior. The analysis of (log) *Number of Fixations* indicated a small preference for the full model, χ^2 (4) = 11.04, $p = 0.026$, which, however, was not confirmed by AIC and BIC, and did not yield an increase in adjusted R^2.
